# Case report: Two siblings with neuronal intranuclear inclusion disease exhibiting distinct clinicoradiological findings

**DOI:** 10.3389/fneur.2022.1013213

**Published:** 2022-10-25

**Authors:** Yutao Liu, Li Zeng, Yanpeng Yuan, Yangyang Wang, Kai Chen, Yan Chen, Jiaying Bai, Feng Xiao, Yuming Xu, Jing Yang, Song Tan

**Affiliations:** ^1^Department of Neurology, The First Affiliated Hospital of Zhengzhou University, Zhengzhou University, Zhengzhou, China; ^2^Key Laboratory of Cerebrovascular Disease of Henan Province, Zhengzhou University, Zhengzhou, China; ^3^Henan Medical Key Laboratory of Neurogenetic and Neurodegenerative Disease, Zhengzhou, China; ^4^Department of Neurology, Sichuan Provincial People's Hospital, University of Electronic Science and Technology of China, Chengdu, China; ^5^Department of Neurology, The People's Hospital of Jianyang City, Jianyang, China; ^6^Sichuan Provincial Key Laboratory for Human Disease Gene Study, Chengdu, China

**Keywords:** neuronal intranuclear inclusion disease, mitochondrial encephalopathy, ribbon sign, *NOTCH2NLC* gene, siblings

## Abstract

Neuronal intranuclear inclusion disease (NIID) is a rare neurodegenerative disorder characterized by the presence of eosinophilic hyaline intranuclear inclusions. Owing to its widely varying clinical manifestations, NIID is frequently misdiagnosed or overlooked. However, a characteristic high-intensity corticomedullary junction signal on diffusion-weighted imaging (DWI) is often indicative of NIID. In this study, we described the case of two sisters with NIID who presented with distinct symptoms and imaging data. The younger sister showed symptoms similar to those of mitochondrial encephalopathy, with a reversible high-intensity signal from the cortex on T2 and DWI. The elder sister showed a characteristic high-signal “ribbon sign” in the corticomedullary junction on DWI. Skin biopsy confirmed that both had neuronal intranuclear inclusion. Two years later, the younger sister also developed the characteristic high-signal “ribbon sign” in the corticomedullary junction on DWI. This case study provides new insights into the complexity of NIID. The findings suggest that patients with this condition, including those belonging to the same family, may exhibit varying clinical and imaging features at different times.

## Introduction

Neuronal intranuclear inclusion disease (NIID) is a gradually progressing neurodegenerative condition characterized by the presence of tissue-wide eosinophilic intranuclear hyaline inclusions in cells of the central and peripheral nervous systems (1). Given its varying clinical features and pathological findings, NIID is considered a heterogeneous disease, although a persistent, highly intense corticomedullary junction on diffusion-weighted imaging (DWI) is generally considered to be a characteristic of this disease (2). Eosinophilic intranuclear inclusion bodies found in skin biopsy samples can also be indicative of NIID (3), and the disease can be confirmed by determining whether GGC repeats are present in the *NOTCH2NLC* gene (4, 5). In this study, we reported two siblings with adult-onset NIID and described their clinical, imaging, and pathological features. The imaging features in our initial patient's older sister provided important clues toward the younger's diagnosis. Two years later, the younger sister also began to exhibit typical hyper-intense signals in the corticomedullary junction on DWI. In particular, we demonstrated that the varying symptoms and imaging features we observed may present a challenge for accurate diagnosis. Our case is unique because none of the cases reported that the patient had typical MRI findings of mitochondrial encephalopathy in the early stage, and later had high signal in the corticomedullary junction following up for several years. This imaging change was discovered and reported for the first time by our group.

## Case description

**Figure 1:**
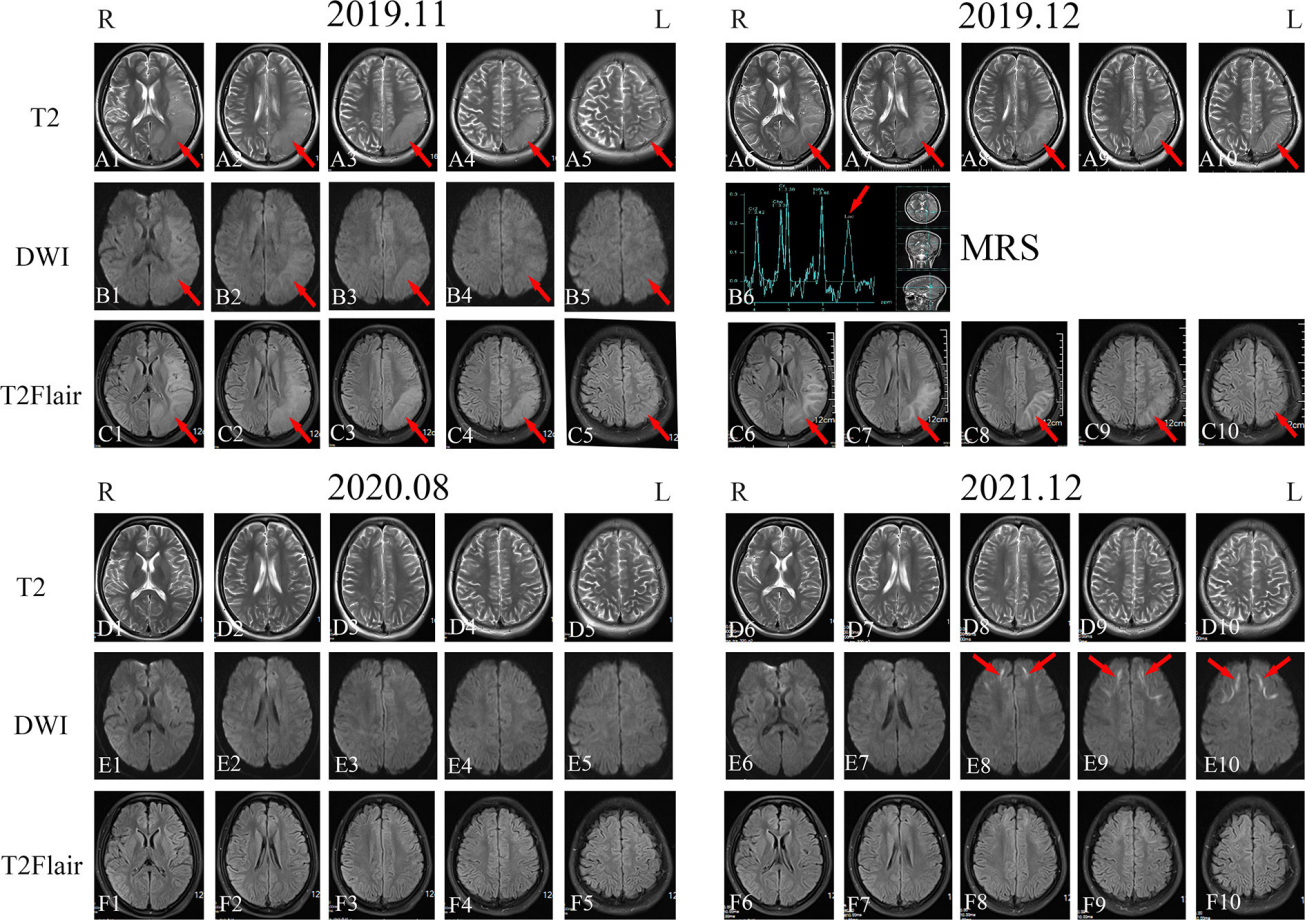
Imaging examination of patient 1. Head magnetic resonance imaging (MRI) in November 2019 showed high-signal intensity in the left temporal, parietal, and occipital lobes on T2-weighted imaging, diffusion-weighted imaging (DWI), and T2 fluid-attenuated inversion recovery (FLAIR) sequences [**(A1–A5)**, **(B1–B5)**, and **(C1–C5)**; red arrows]. MRI in December 2019 showed high-signal intensity in the left temporal, parietal, and occipital lobes on T2-weighted imaging, magnetic resonance spectroscopy, and T2 FLAIR sequences [**(A6–A10)**, **(B6)**, and **(C6–C10)**; red arrows]. Head MRI in August 2020 revealed that the high-intensity signal on the left temporal, parietal, and occipital lobes on T2-weighted imaging, DWI, and T2 FLAIR sequences had disappeared [**(D1–D5)**, **(E1–E5)**, and **(F1–F5)**]. Head MRI in December 2021 showed bilateral and relatively symmetrical ribbon signs with high-intensity signals along the corticomedullary junction of the frontotemporal-parietal lobes on DWI [**(D6–D10)**, **(E6–E10)**, and **(F6–F10)**; red arrows].

A 20-year-old woman (patient 1) visited our center after having experienced recurrent headache, blurred vision, and paroxysmal partial body numbness and weakness for 6 years. The patient's initial symptoms in 2016 consisted of right-side numbness from the calf to the upper limb and face that improved after 10 min and disappeared after 1 h. Several months later, the patient experienced blurring in her right visual field with a headache that disrupted sleep throughout the night. By the next morning, the patient's vision had cleared, while the severity of their headache had alleviated. Brain computed tomography (CT) and electroencephalography (EEG) showed normal results. In November 2019, the patient experienced another attack with paroxysmal right limb and face numbness and weakness accompanied by hemianopia, vomiting, and confusion, wherein the patient lost consciousness for 3 days. Later that month, the patient's electromyography (EMG) showed that motor and sensory nerve conduction velocities in her limbs were slower than normal. The F-wave conduction velocity was also slow. The motor unit potential time range of the detected muscles was normal, while the amplitude of the wave was high, risking neurogenic damage. Overall, obvious demyelination of peripheral nerves in four limbs was present. Brain magnetic resonance imaging (MRI) showed swelling of the gyri and shallow sulci in the left cerebral hemisphere, especially in the temporoparietal-occipital lobe, on T2, DWI, and T2 fluid-attenuated inversion recovery (FLAIR) sequence in November 2019 (Figures 1A1–A5,B1–B5,C1–C5); the signal was slightly higher in December 2019 (Figures 1A6–A10,C6–C10). Magnetic resonance spectroscopy (MRS) performed in December 2019 showed that a broad lactic acid peak was observed (Figure 1B6). Lactic acid exercise tests yielded elevated blood levels at baseline (3.5 mmol/L; reference: 0.5–2.2 mmol/L), after running for 15 min (8.3 mmol/L; three times higher than normal), and 10 min after completing the run (4.9 mmol/L; twice the normal level); hence, mitochondrial myopathy was suspected. The patient's liver and renal functions, electrolytes, trace element levels, myocardial enzymes, blood ammonia, thyroid function, tumor marker levels, hepatitis B test results, and HIV test results were normal. Antibody tests for autoimmune and paraneoplastic disorders were also negative on serum and cerebrospinal fluid analysis. Video EEG showed moderately abnormal, single-scattered sharp waves; additionally, sharp slow waves were observed in the frontal, parietal, and temporal areas on both sides. The patient was discharged after her symptoms improved. During the months after discharge, the patient experienced paroxysmal partial body numbness on three occasions, accompanied by difficulty speaking or by headache. As these symptoms all resolved within 5 min, the patient did not consult a physician. In August 2020, the patient returned to the hospital for re-examination, despite not experiencing any discomfort at the time. Physical examination revealed that the patient's memory had declined; cognitive screening tests showed that the patient's Mini-Mental State Examination (MMSE) score and Montreal Cognitive Assessment (MoCA) score were 24/30 and 20/30, respectively. Brain MRI showed no abnormalities in the sulci or gyri, and no diffusion limitation was found on DWI (Figures 1D1–D5,E1–E5,F1–F5). EMG showed symmetrical multiple peripheral nerve damage in the limbs as well as severe demyelination; the patient's muscles also exhibited neurogenic damage.

Given the chronic course of the central and peripheral nervous system defects, the patient's disease apparently began manifesting at approximately 20 years of age following poor exercise tolerance and fatigue experienced since childhood. Brain MRI showed laminar necrosis of the left temporo-occipital lobe, although the abnormality disappeared completely after 9 months. Given that the patient's aforementioned test data highly suggested mitochondrial encephalomyopathy, muscle biopsy and mitochondrial-related gene analysis were performed; however, both tests showed no abnormalities.

**Figure 2:**
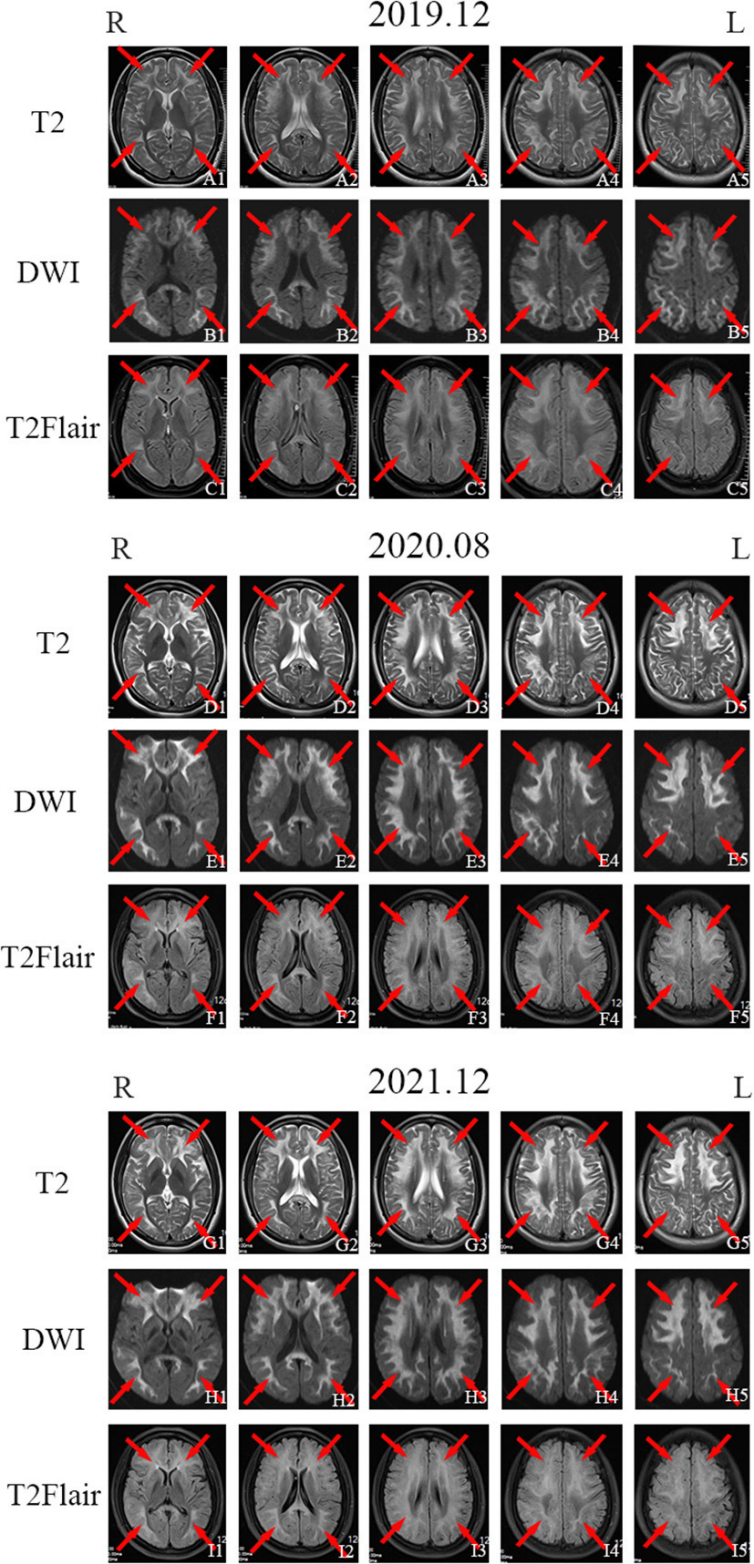
Imaging examination of patient 2. Head magnetic resonance imaging (MRI) in November 2019 showed bilateral and relatively symmetrical ribbon signs with high-intensity signals along the corticomedullary junction of the frontotemporal-parietal lobes and corpus callosum on T2-weighted imaging, diffusion-weighted imaging (DWI), and T2 fluid-attenuated inversion recovery (FLAIR) sequence [**(A1–A5)**, **(B1–B5)**, and **(C1–C5)**; red arrows]. Head MRI in August 2020 [**(D1–D5)**, **(E1–E5)**, and **(F1–F5)**; red arrows] and December 2021 [**(G1–G5)**, **(H1–H5)**, and **(I1–I5)**; red arrows] showed the same high signal along the corticomedullary junction of the frontotemporal-parietal lobes and corpus callosum on T2-weighted imaging, DWI, and T2 FLAIR sequences as were observed in November 2019.

The patient's sister (patient 2) was 32 years old and had experienced symptom onset at 27 years of age; symptoms included recurrent headache with blurred vision and progressive contralateral limb numbness and weakness. At the onset of symptoms for 5 years before (in 2017), the patient's episodes occurred once in every 2–3 months and the symptoms improved significantly within 2–3 days. An EMG performed at West China Hospital in December 2017 showed peripheral neurogenic damage in the limbs with marked effects on sensory and motor nerve conduction. Brain MRI showed abnormal signals bilaterally in the frontotemporal cortex and corpus callosum. EEG results were mildly abnormal, with an increase in slow waves in each lead on both sides. The patient was suspected to have NIID. On 27 November 2019, the patient experienced another headache as well as blurred vision in the right eye followed by vomiting and an inability to speak; these symptoms did not improve with anti-headache drugs. The patient subsequently performed second brain MRI on 5 December 2019, which showed extensive and slightly elongated T1 shadows, as well as a slightly elongated T2 signal shadow, bilaterally in the frontal, temporal, parietal, and occipital cortices and corpus callosum. Moreover, T2 FLAIR and DWI signals were high (Figures 2A1–A5,B1–B5,C1–C5). Lactic acid was within a normal range (1.6 mmol/L). The frequency of the patient's headache began to increase in March 2020, with several monthly attacks occurring. In August 2020, the patient's physical examination revealed that her memory and cognition had declined and that she exhibited signs of ataxia. On cognitive-screening testing, the patient's MMSE and MoCA scores were 27/30 and 21/30, respectively. Brain MRI showed symmetrical ribbon-like elongated T1 and slightly elongated T2 signals in the corticomedullary junction and corpus callosum of both cerebral hemispheres. T2 FLAIR showed a high signal, and the apparent diffusion coefficient slightly decreased; no enhancement was observed in the lesions (Figures 2D1–D5,E1–E5,F1–F5).

A review of patient 1's personal history revealed that she had exhibited poor performance in mathematics and sports (jump and long-running) since childhood; moreover, the patient was sensitive to pungent odors and coughed easily. The patient 1 (II-2)'s family history showed her mother (I-2) also had a long history of repeated severe headache and died at the age of 49 due to “headaches and disturbances in consciousness” without genetic testing. Her father (I-1) had no similar symptoms and was ruled out by genetic testing. Her old sister (patient 2, II-1) had symptoms that included recurrent headache with blurred vision and progressive contralateral limb numbness and weakness (Supplementary Figure 1).

**Figure 3:**
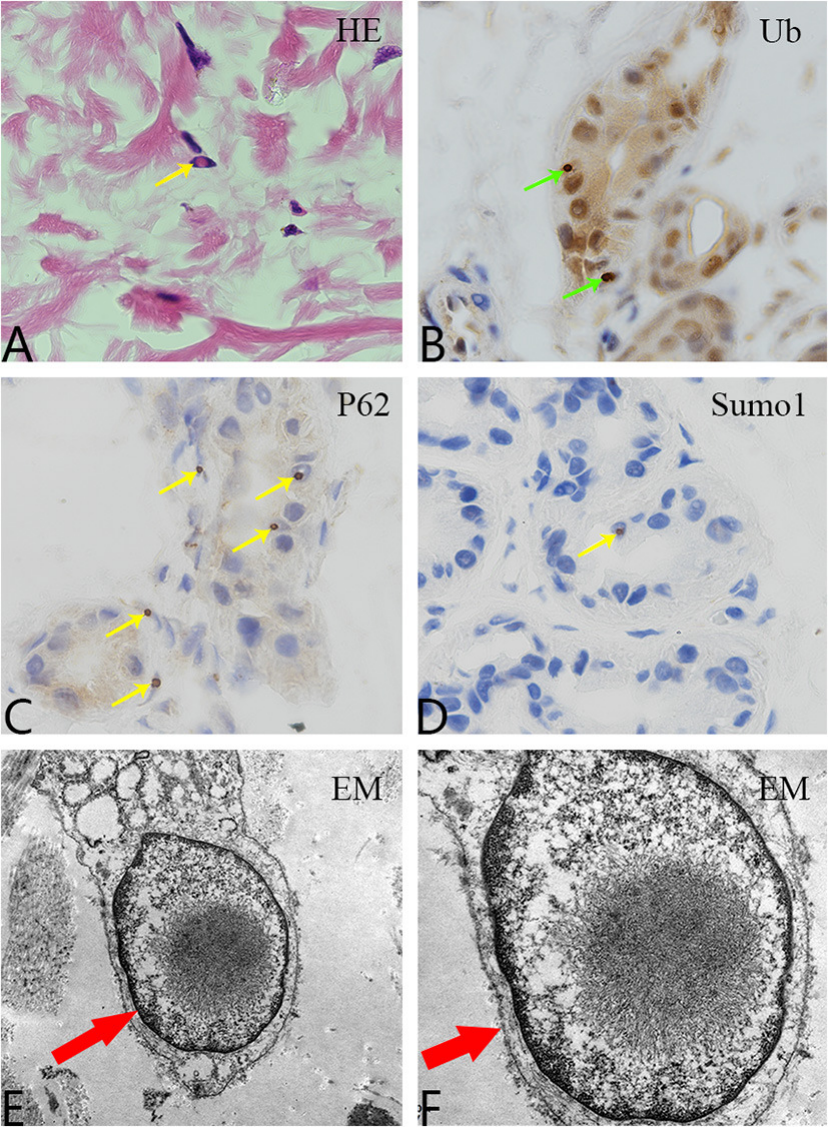
Pathological examination. **(A)** Hematoxylin and eosin staining shows eosinophilic inclusion bodies in some skin fibroblasts (100×, yellow arrow). **(B)** Anti-ubiquitin immunohistochemical staining. Inclusion bodies are visible in the nuclei of some skin sweat gland cells (100×, green arrow). **(C)** Anti-P62 immunohistochemical staining. Inclusion bodies are observed in the nuclei of some sweat gland cells (100×, yellow arrow). **(D)** Anti-SUMO1 immunohistochemical staining. Inclusion bodies are visible in the nuclei of some sweat gland cells (100×, yellow arrow). **(E,F)** Electron microscopy revealed a circular inclusion body structure in the nucleus of a sweat gland cell (15,000× and 30,000×, respectively; red arrows).

**Figure 4:**
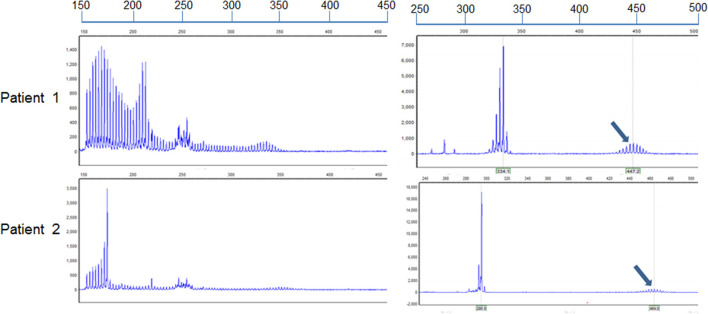
Identification of alleles with GGC repeat expansions in the two sisters. Repeat-primed PCR and fluorescence amplicon length analysis of both patients showed GGC repeat expansions. The number of repeats was determined by comparing the length of the highest signal peak of the expanded allele (arrows) to that of the human reference gene sequence as previously described (6). The formula to determine repeat size was ([“length of the highest signal peak of the expanded allele” bp – 185 bp] / 3 bp + 13). The assumed length of the PCR product was 185 bp in accordance with the standardized GRCh38.p13 human genome, containing the reference (GGC)9(GGA)2(GGC)2 sequence (*n* = 13).

Both sisters underwent skin biopsy. Light and electron microscopy showed eosinophilic, ubiquitin-positive, p62-positive, and SUMO-1-positive intranuclear inclusions in the adipocytes, fibroblasts, and sweat gland cells in both patients (Figures 3A–F). Both sisters were also tested for the *NOTCH2NLC* pathogenic gene of NIID; patients 1 and 2 had 70 and 66 GGC repeats, respectively (Figure 4), suggesting NIID. A genetic test for cerebral autosomal dominant arteriopathy with subcortical infarcts and leukoencephalopathy was negative, and subsequent genetic analysis of the fragile X chromosome mental retardation gene 1 showed normal GGC repeat numbers in both sisters, which ruled out fragile X-related tremor/ataxia syndrome. Given these clinical findings, both sisters were diagnosed with NIID.

Between her discharge in 2020 and the year after, patient 1 had another episode of right hemiparesis with a contralateral headache that was less severe than before. In December 2021, the patient returned to the hospital for re-examination; cognitive screening tests showed that her MMSE (27/30) and MoCA (25/30) scores were both higher than those in 2020. Brain MRI in December 2021 showed bilateral corticomedullary junction white matter areas in the frontal lobes that were patchy with slightly elongated T1 and T2 signal shadows. T2 FLAIR showed a slightly elevated signal, while diffusion was limited on the DWI sequence (Figures 1D6–D10,E6–E10,F6–F10). Lactic acid levels in the blood were normal (2.2 mmol/L) at baseline. EMG showed that extensive peripheral nerve lesions and axonal myelin sheath damage were present, especially in the lower limbs.

Since October 2021, the frequency of patient 2's headache had increased to once a week with nausea and vomiting. On 30 November 2021, the patient's cognitive screening tests showed MMSE and MoCA scores of 27/30 and 22/30, respectively. Brain MRI in December 2021 revealed minimal change from August 2020 (Figures 2G1–G5,H1–H5,I1–I5). EMG showed symmetrical peripheral nerve damage (mainly demyelination) in the limbs, including both motor and sensory nerves.

Both patients' clinical features are shown in Supplementary Table 1.

## Discussion

Neuronal intranuclear inclusion disease is a chronic progressive neurodegenerative disease characterized by transparent eosinophilic inclusions in the central and peripheral nervous systems and internal organs (2, 3). Brain images of patients with adult-onset NIID exhibit characteristic features (4); DWI shows a high-intensity signal at the corticomedullary junction known as the “ribbon sign” (2, 5). Histopathologically, adult-onset NIID is characterized by nuclear inclusions in skin cells, constituting the main diagnostic criterion for this condition (1, 7). GGC repeat expansion in the 5′UTR region of the human-specific *NOTCH2NLC* gene is the genetic underpinning of NIID (8, 9). While the number of GGC repeats normally does not exceed 40, patients with NIID typically present with a minimum of 60 repeats (6, 10). Some studies have also demonstrated that mitochondrial encephalomyopathy is a characteristic of NIID (11, 12). The presentations observed in our patients offer additional evidence regarding the clinical signs of this disease and help clarify the fact that mitochondrial diseases that lack accompanying pathological manifestations may be indicative of NIID.

The two patients in our study were sisters aged 14 and 27 years at the time of the onset of their diseases. Their symptoms indicated that they belonged to the limb-weakness subgroup of familial adult-onset NIID. According to previous studies, the main symptom of this disease is limb weakness in young people and memory loss in older individuals (13, 14). However, our patients developed cognitive impairment at a young age. Brain MRI analysis revealed reversible laminar necrosis of the cortex with no ribbon sign in patient 1, which is consistent with the imaging features of mitochondrial encephalomyopathy. In contrast, the DWI sequence of the head MRI of patient 2 showed the characteristic ribbon sign; these differences highlight the clinical heterogeneity of the disease. Even if the clinical manifestations of patients in the same family are similar, their imaging findings may be considerably different. The clinical manifestations, imaging features, and laboratory tests of patient 1 were highly indicative of mitochondrial encephalomyopathy; however, muscle pathology or genetic testing did not support this conclusion, thereby complicating the diagnosis. After performing a detailed evaluation of patient 2, we observed the characteristic ribbon sign on imaging that prompted further investigation of the possibility of NIID. Finally, skin biopsy and genetic testing confirmed the diagnosis of NIID in both sisters. Within 2 years of follow-up, patient 1 also began to exhibit typical hyper-intense signals in the corticomedullary junction on DWI; this indicated that the patients had different imaging features that changed over time. A DWI high signal at the junction of the cortex and medulla may be a typical manifestation of progression to the late stage of the disease. Different imaging manifestations characterize the early stage of the disease, such as those observed in mitochondrial encephalopathy in patient 1. Furthermore, a highly consistent ribbon sign tends to appear in the late stage. Moreover, in patient 1, the gyrus of the left cerebral hemisphere was swollen, and the sulci became shallow, which was obvious in the parietal-temporal-occipital lobe and similar to mitochondrial performance. However, the high signal in the corticomedullary junction area in the later stage began from the frontal lobe. Whether this typical imaging change also developed from front to back needs to be confirmed during follow-up visits. Patient 2 had a typical DWI high signal at the junction of the cortex and medulla more than 2 years after onset and continued to exhibit typical imaging manifestations until 5 years after the course of the disease. An imaging observation study that tracked a patient for 16 years found that the patient's initial T2 FLAIR images showed spatial high-intensity lesions in the subcortical white matter, while DWI showed no specific abnormalities (15). It took 16 years to develop leucoencephalopathy on T2 FLAIR images, and the high-intensity signal in the corticomedullary junction on DWI had gradually expanded after symptom onset. This is also an important reason why the disease is prone to oversight and misdiagnosis.

The number of GGC repeats is approximately 60 in patients with tremors, 80 in those with Parkinson's disease, 120 in those with cognitive impairment, and 200 in those with limb weakness. Patients 1 and 2 had 70 and 66 GGC repeats, respectively, but had symptoms of limb weakness and cognitive impairment; this ought to prompt additional exploration of the relationship between GGC repeats and clinical phenotypes. The correlation between the repetition times and clinical manifestations of other polynucleotide repeat diseases should also be considered. For example, ankylosing muscular dystrophy and ophthalmo-pharyngeal muscular dystrophy have similar relations with repetition times.

## Conclusions

The two sisters presented with distinct clinical manifestations and imaging characteristics. By analyzing the images of patient 2, we were able to determine the diagnosis of patient 1. Overall, this case study provides new information regarding our understanding of NIID.

## Data availability statement

The original contributions presented in the study are included in the article/Supplementary material, further inquiries can be directed to the corresponding author/s.

## Ethics statement

Written informed consent was obtained from the individual(s) for the publication of any potentially identifiable images or data included in this article.

## Author contributions

YL drafted and revised the manuscript and analyzed and interpreted the data. LZ performed skin biopsy, muscle pathology analysis, muscle pathology photography, and clinical evaluation. YY performed skin pathology analysis and photography. YW performed gene detections. KC performed a muscle biopsy. YC recommended patients, conducted partial examinations, and collected medical history. JB collected medical history and blood specimens. FX collected medical history. YX and JY performed a critical revision of the manuscript. ST collected family clinical data, performed the clinical evaluation and preliminary diagnosis, revised the manuscript, and supervised the study. All authors contributed to the article and approved the submitted version.

## Funding

This study was supported by the Department of Science and Technology of Henan Province (Social Development, Grant No. 212102310825) to YL, the National Natural Science Foundation of China (Grant Nos. U1904207 and 91849115) and the National Key R&D Program of China (Grant No. 2017YFA0105003) to YX, the Provincial and Ministry of Health Construction Committee of Henan Province (SB201902012), Henan Young and Middle-Aged Health Science and Technology Innovation Talent Training Project—Outstanding Young Talents to JY, the Sichuan Provincial People's Hospital (Grant No. 2017QN12), the Sichuan Provincial Science and Technology Department (Grant No. 2018JY0160) to YX, and the National Natural Science Foundation of China (Grant No. 81501026) to ST.

## Conflict of interest

The authors declare that the research was conducted in the absence of any commercial or financial relationships that could be construed as a potential conflict of interest.

## Publisher's note

All claims expressed in this article are solely those of the authors and do not necessarily represent those of their affiliated organizations, or those of the publisher, the editors and the reviewers. Any product that may be evaluated in this article, or claim that may be made by its manufacturer, is not guaranteed or endorsed by the publisher.
