# Supplementary figures and images for: Case report: Two siblings with neuronal intranuclear inclusion disease exhibiting distinct clinicoradiological findings
Source: Front Neurol. 2022 Oct 25;13:1013213. doi: 10.3389/fneur.2022.1013213 (PMC9642335; doi:10.3389/fneur.2022.1013213)

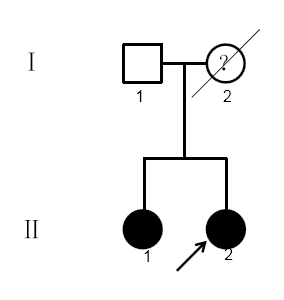

Supplement: Supplementary file 1 [file Image_1.TIF]
