# Supplementary material for: Case report: Two siblings with neuronal intranuclear inclusion disease exhibiting distinct clinicoradiological findings
Source: Front Neurol. 2022 Oct 25;13:1013213. doi: 10.3389/fneur.2022.1013213 (PMC9642335; doi:10.3389/fneur.2022.1013213)
Supplement: Supplementary file 2 [file Table_1.DOCX]

Supplementary Material

**Supplementary Table 1.** Clinical features of patients with neuronal intranuclear inclusion disease

| **Characteristic** | **Patient No.** | |
| --- | --- | --- |
|  | **1** | **2** |
| Age at onset (years) | 14 | 27 |
| Sex | F | F |
| Disease duration | 6 | 5 |
| Clinical manifestations |  |  |
| Headache | + | + |
| Blurred vision | + | + |
| Dementia | + | + |
| Muscle weakness | + | + |
| Numbness | + | + |
| Ataxia | - | + |
| Irritating dry cough | + | - |
| Vomiting | + | + |
| Sensory disturbance | + | - |
| Transient visual field abnormality | + | + |
| Transient hemiparesis | - | + |
| Disturbance of consciousness | + | - |
| Cognitive screening test ^a^ |  |  |
| MMSE in August 2020 | 24 | 27 |
| MoCA in August 2020 | 20 | 21 |
| MMSE in December 2021 | 27 | 27 |
| MoCA in December 2021 | 25 | 22 |
| MRI findings |  |  |
| High-intensity signal along the cortico-medullary junction on DWI (2019) | - | + |
| High-intensity signal along the cortico-medullary junction on DWI (2020) | - | + |
| High-intensity signal along the cortico-medullary junction on DWI (2021) | + | + |
| Diffuse high-intensity signal of cerebral white matter on FLAIR images (2019) | + | - |
| Diffuse high-intensity signal of cerebral white matter on FLAIR images (2020) | - | - |
| Diffuse high-intensity signal of cerebral white matter on FLAIR images (2021) | - | - |
| Electromyography |  |  |
| Neurogenic damage in peripheral nerve (2019) | Demyelination | Demyelination |
| Neurogenic damage in peripheral nerve, mainly demyelination (2020) | Mainly demyelination | Demyelination |
| Neurogenic damage in peripheral nerve (2021) | Demyelination with axonal damage | Mainly demyelination |
| Electroencephalogram |  |  |
| Abnormal (2019) | - | - |
| Abnormal (2020) | - | - |
| Abnormal (2021) | Borderline abnormal | - |
| Blood lactic acid level elevated (2019) | + | - |
| Blood lactic acid level elevated (2020) | - | - |
| Blood lactic acid level elevated (2021) | - | - |

^a^The MMSE score was considered decreased below a cutoff of 24, and the MoCA score was considered decreased below a cutoff of 26.

DWI, diffusion-weighted imaging; F, female; FLAIR, fluid-attenuated inversion recovery; MMSE, Mini-Mental State Examination; MoCA, Montreal Cognitive Assessment; MRI, magnetic resonance imaging.
