# Supplementary material for: Case report: Two siblings with neuronal intranuclear inclusion disease exhibiting distinct clinicoradiological findings
Source: Front Neurol. 2022 Oct 25;13:1013213. doi: 10.3389/fneur.2022.1013213 (PMC9642335; doi:10.3389/fneur.2022.1013213)
Supplement: Supplementary file 3 [file Table_2.DOC]

Supplement Figure 1. Family with abnormal GGC repeats in *NOTCH2NLC* gene mutations.

Proband is indicated with an arrow. Solid symbols represent affected individuals, open symbols represent unaffected individuals. Squares represent men. Circles represent women. Lines through symbols represent deceased individuals.
